# Exploring the culturally sensitive sexual and reproductive health information communication skill needs of parents in Ghana

**DOI:** 10.4102/phcfm.v15i1.4101

**Published:** 2023-10-20

**Authors:** Frank B. Agyei, Doreen K. Kaura, Janet D. Bell

**Affiliations:** 1 Department of Nursing and Midwifery, Faculty of Medicine and Health Sciences, Stellenbosch University, Tygerberg, South Africa

**Keywords:** parents, adolescents, sexual and reproductive health, culture, information, communication, intervention, motivation

## Abstract

**Background:**

Parents play a vital role in the sexual and reproductive health (SRH) of adolescents. Parents’ communication with their adolescents regarding SRH is considered an important part of adolescent development, as this contributes to optimising safe SRH.

**Aim:**

This phase of the study explored the culturally sensitive SRH information communication skill needs of parents, based on their personal and social motivation, within the Ghanaian context.

**Setting:**

The study was conducted at the Asante Akyem North Municipality of Ghana.

**Methods:**

This article describes the second phase of an explanatory, sequential, mixed-method study. Following on from the first phase systematic review, this second phase comprised a qualitative descriptive study where 10 purposively sampled parents of adolescents participated in semi-structured interviews to elicit data. Braun and Clarke’s thematic data analysis process was applied. Data were saved and managed in Atlas.ti (version 23.0.7).

**Results:**

Four themes emerged on communication skills: SRH information, parent and adolescent factors, contextual factors and communication skill needs. Parent and adolescent communication on SRH takes place occasionally. Parents lack the skills to communicate with adolescents regarding SRH.

**Conclusion:**

Parents in this context require skills to communicate SRH information with their adolescent children. A culturally appropriate intervention that supports SRH information communication between parents and adolescents may have value in guiding this communication process.

**Contribution:**

The findings of this study can contribute to the adaptation of a culturally sensitive SRH information communication intervention in Ghana which will promote adolescent SRH.

## Introduction

The sexual and reproductive health (SRH) of adolescents calls for global concern, as risky adolescent sexual behaviours can have significant health-related consequences. Risky behaviours include early sex initiation, unprotected sexual intercourse, indiscriminate sex with numerous sexual partners and alcohol-induced sexual intercourse.^1^ Such behaviours can have consequences such as unsafe abortions, sexually transmitted infections (STIs), pregnancy and childbirth-related complications, among others.^2^ This being the case, adolescents require reliable SRH information to mitigate these challenges.

Adolescents usually get SRH information from their peers, teachers, parents and the internet.^3^ Literature underscores the fact that the family, and in particular parents, play a formidable role in adolescents’ acquisition of SRH information.^4^ This means that parents play a significant role in the SRH behaviours of adolescents. Therefore, it is significant for parents and adolescents to communicate on SRH issues. Sexual and reproductive health information communication is how parents and adolescents share values and knowledge on SRH.^5^ Effective communication between parents and adolescents regarding SRH information has been emphasised as a factor that may influence adolescents to embrace safer sexual behaviour, thereby reducing sexual health concerns.^6,7,8,9^ Consequently, parents’ SRH communication may prevent or reduce sexual behaviours associated with morbidity and mortality.^10^ Negative sexual behaviours and practices, such as teenage pregnancy, increase where SRH communication is absent or poor.^11^ Despite this, it has been identified that, globally, parents face personal, communal and cultural barriers which are predictors of poor SRH communication between themselves and adolescents.^12^

Converging evidence for Africa reveals that communication between parents and adolescents on SRH is poor due to these barriers.^10,11,13,14^

In Ghana, findings from a study noted that, although most parents had at some point in time discussed SRH with their adolescents, the discussions centred on only a few topics.^15^ Adolescents do not go to their parents for SRH information because of a lack of confidence and trust, a lack of parental availability, inappropriate language and peer norms. This could be due to parents’ lack of SRH knowledge and communication skills.

A study confirmed that having adequate SRH knowledge as a parent is central to building the trust and confidence required for effective parent-adolescent SRH communication.^16^

Despite effective SRH education being necessary for the healthy sexual development of adolescents, limited parental self-efficacy as well as cultural norms create an uncomfortable atmosphere for parents to discuss issues of SRH with adolescents in African countries^9^ such as Ghana. This is considered a major barrier. Some of the elements that have emerged as barriers for parents include a lack of knowledge about how to initiate conversations about SRH, a lack of correct information or knowledge of the correct language to use in local vernacular to describe anatomy or discuss explicit sexual topics, and the absence of communication skills to talk about the delicate subject of SRH with an adolescent.^9^ Parents lacked confidence to discuss SRH, stating that their adolescent was better educated and more knowledgeable and experienced in sexual health issues.^15,17^

The need for interventions to train and assist parents and adolescents to communicate SRH information and values has been highlighted in literature.^13,18^ Sexual and reproductive health information and communication interventions have worked in other contexts, more especially in Europe and Asia.^1,19^

The growing evidence of poor SRH communication between parents and adolescents in Africa, and specifically Ghana, presents an opportunity for a culturally appropriate SRH information communication intervention for parents and adolescents to be developed. To achieve this, the information and communication skill needs of parents and adolescents in the Ghanaian context must be explored and considered in the intervention development. This article therefore describes the SRH information and communication skill needs of parents, based on their personal and social motivation, in the Ghanaian context.

This phase of the study is a follow-up on a systematic review in phase one of the study, which found that SRH information communication improves when the skill to communicate such information is sufficient. The skill could also be influenced by factors such as the information to communicate and the attitudes of the individual towards communication, together with social norms. This article, which reports the qualitative phase of the study, highlights the culturally sensitive SRH information communication skill needs of parents in Ghana.

### Theoretical framework

**FIGURE 1:**
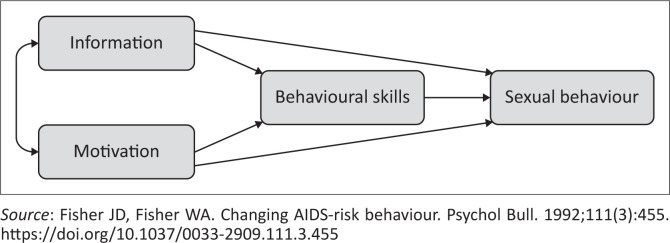
Original information, motivation and behavioural skills model.

The researcher made use of the information, motivation and behavioural (IMB) skills model^20^ to underpin the study (Figure 1)^21^. The IMB skills model assumes that health-related information, motivation and behavioural skills are the basic determinants of performance of health behaviours.^21^ This is to say that, when people have relevant information and sufficient personal and social motivation, they will have the skill necessary to enable a healthy behaviour. In the study, it was assumed that if parents have SRH information, are well motivated and have the skills, they are likely to communicate SRH information with their adolescents. The model was used as a lens for data analysis, to explain the culturally sensitive SRH communication skills of parents of adolescents.

## Research methods and design

### Study design

This is the second phase of an explanatory sequential study. The first phase was a systematic review that was conducted to identify effective interventions to improve SRH information communication in lower-and-middle-income countries (LMICs). Qualitative description was employed for this phase of the study.^22,23^ Qualitative description is a label used for qualitative studies which are descriptive in nature, particularly those examining health-related phenomena. The design is built on the general principles of naturalistic inquiry.^22,23^ In this design, it is possible for the analysis of data to remain true to the voice of participants and contribute to the transparency of the researcher’s interpretations.^24^

### Study setting

The study was conducted at the Asante Akyem North Municipal District of Ghana. Residents of this district include almost all the ethnic groups in Ghana, and this fosters ethnic and cultural diversity. The Akan culture is dominant. However, the migrants from other regions of the country practice their culture alongside Akan or Asante culture. English and ‘Asante Twi’ languages are the main languages for communication in the municipality.^25^

### Study population and sampling strategy

Information flyers distributed at a community centre provided potential participants (parents of adolescents [13–16 years]) with information and contact details relevant to the study. For the purposes of this study, a parent was defined as a biological mother or father, or a male or female guardian, of an adolescent within the specified years. To capture the nuances of different developmental needs, the researcher included five parents of adolescents aged 13–14 and five parents of adolescents aged 15–16; similarly, to ensure variability among parents, a minimum of three males and three females were sampled. This age group of adolescents was considered because it represents a transition from early to late adolescence. Middle adolescents have experienced the characteristics of early adolescence, and late adolescents are at (or almost at) the age where they can give legal consent and may not depend on their parents for SRH decisions. Adolescents of these parents were interviewed in another phase of the study. Parents whose adolescents were married or cohabiting were excluded from the study. The purposive sampling approach was used to select parents who were willing to participate in the study. Parents who were interested contacted the researcher and his assistants. The research team followed up to discuss the study with the parents who were willing, whose consent was given. A mutually agreeable date, time and venue were scheduled. Before commencement of interviews, a consent form was signed. On data saturation, the final study sample comprised 10 participants.

### Data collection

An interview guide was used in face-to-face, semi-structured, individual interviews. The interview guide was developed based on the IMB model and on the findings from the systematic review of effective SRH information communication interventions in LMICs, which was conducted in phase one of this study. Parents were asked questions on the SRH information they communicate with their adolescents, their motivation to communicate such information and their skill needs. The interview guide was developed in English and was translated into the ‘Asante Twi’ language and back translated to prevent loss of contextual meaning. The interviews occurred at a time and place convenient to the participant. The interviews which lasted between 35 and 60 min were audio-recorded, with the permission of participants. Field notes were taken to capture additional information. Data collection was done from August 2022 to January 2023.

Reflexivity was ensured throughout the process. The researcher documented the comments of participants and his thoughts. The researcher’s insight into, and interpretation of, the data was documented. This helped the researcher to understand the data and assist in communicating the findings.

### Data analysis

All interviews were transcribed verbatim in the language in which they were undertaken. Transcripts in the Akan language were then translated verbatim into English. To ensure accurate transcription, each transcript was checked against the recorded interview. The transcribed data were then uploaded into Atlas.ti software (version 23.0.7). Thematic analysis of the data was done inductively, in six phases, by the researcher and two assistants.^26^ Firstly, the researcher and his assistants familiarised themselves with the data and paid attention to little details. Secondly, initial codes were then generated by organising data into meaningful units and afterwards combined to form groups of themes. The themes were reviewed by the researcher and his supervisors and categorised into subthemes. Field notes were also reviewed and added to the information obtained. Conclusions were drawn from the identified categories and the themes to meet the objectives. The themes were defined and named, and then the report was produced.

### Ethical consideration

The Health Research Ethics Committee (HREC) of the Stellenbosch University approved the study with reference number S21/08/159 on 06 January 2022, and renewal and extension were further granted on 06 January 2023. Following this approval, the Committee on Human Research, Publication and Ethics of the College of Health Sciences of the Kwame Nkrumah University of Science and Technology granted approval in Ghana with reference number CHRPE/AP/356/22 on 19 July 2023. Participants were given detailed information on the study and were informed that participation was entirely voluntary. They were assured of confidentiality, and they voluntarily signed the consent form before the interviews were conducted.

## Results

### Characteristics of parents

Ten parents of adolescents took part in the study. Four of them were male and six were female. Other characteristics are detailed in Table 1.

**TABLE 1:** Participants’ characteristics.

Participant	Age (years)	Gender	Age of adolescent (years)	Marital status	Number of children	Level of education	Occupation	Tribe	Language	Religion
P1	38	F	15	Married	3	Primary	Cleaning service	Ashanti	Asante Twi	Christianity
P2	42	F	16	Married	3	No formal education	Trading	Frafra	Frafra, Asante Twi	Islamic
P3	76	F	14	Married	4	No formal education	Farming	Ashanti	Asante Twi	Christianity
P4	42	M	14	Married	3	Tertiary	Businessman	Akuapem	English, Akuapem Twi	Christianity
P5	39	F	13	Married	4	Tertiary	Teaching	Ashanti	English, Twi	Christianity
P6	36	F	13	Married	3	Tertiary	Physician assistantship	Ashanti	English, Twi	Christianity
P7	40	M	15	Married	3	Tertiary	Nursing	Fanti	English, Fante Twi	Christianity
P8	48	M	13	Married	4	Tertiary	Teaching	Ashanti	English, Asante Twi	Christianity
P9	63	M	16	Married	3	Tertiary	Administrator	Anlo Ewe	English	Christianity
P10	50	F	16	Divorced	3	Tertiary	Lab technician	Dagomba	English, Twi, Hausa	Islamic

### Themes generated from the study

Although the culturally sensitive SRH information communication skill needs of parents were the focus of the exploration, it emerged that there is a set of interrelated factors that influence the communication skills needs of parents. Four themes emerged with corresponding subthemes (Table 2).

**TABLE 2:** Themes and subthemes.

Themes	Subthemes
SRH information communicated	SRH topics discussed by parents
	Source of SRH information
Parent and adolescent factors	Parental factors
	Adolescent factors
	Parent-adolescent relationship
Contextual factors	Perceived social support
	Cultural norms
SRH communication skill needs of parents	Actual ability to communicate
	Nature of communication

SRH, sexual and reproductive health.

### Sexual and reproductive health information communicated

‘SRH information communicated’ as a theme encompasses the SRH content conveyed by parents to their adolescents. Two subthemes emerged: the SRH topics they discussed with their adolescents and their sources of information.

#### Sexual and reproductive health topics discussed by parents

Sexual and reproductive health topics discussed by parents included changes during adolescence, menstruation and personal hygiene, abstinence, sex, sexual relationships, teenage pregnancy, STIs and virginity. Although these topics were not discussed by all parents in the study, each parent had some topics of interest to discuss.

One participant focused his discussion on the changes adolescents go through in adolescence. He also discussed menstruation and how the adolescent should take care of her personal hygiene during menstruation.

‘The focus was on the changes in the adolescent, the hormonal changes, the physical changes and attitudinal changes … the need for her to keep herself hygienically during menstruation.’ (P8, M, 48 years)

Another participant informed the adolescent about sex, which she considers a ‘no-go’ area until marriage, premarital pregnancies and STIs. The participant esteemed abstinence and so transferred that information to the adolescent.

‘I told her that immediately after her menses she should know that sex is a no-go area till she gets married. Otherwise, she will have premarital pregnancies and STIs that comes after sex.’ (P10, F, 50 years)

The information they shared was based on their knowledge on the various topics. The accuracy of the information communicated may also be due to the sources of information.

#### Sources of information

These are the areas from which the parents derived the SRH information they shared with their adolescents. These sources included books, workshop materials, radio, television, internet, phones, social media, Church, Mosque and personal experiences. A participant shared that:

‘We watch some on television, we use the internet to Google and we have books that we read to find information about all these things.’ (P5, F, 39 years)

Others also depended on their experiences when growing up, because they did not have any formal education and cannot read. This is illustrated in the quote below:

‘It is because we were children before, we had mothers who spoke to us. That is where I learned it from … what I have been through … because I cannot read.’ (P2, F, 42 years)

Some participants also mentioned that they got the information from the Church and the Mosque:

‘At the church, they have been preaching about it.’ (P4, M, 42 years)

‘It is being talked about when the Mallams and Imams are giving sermons.’ (P10, F, 50 years)

This could be why abstinence was the most discussed topic by parents.

### Individual parent and adolescent factors influencing communication

These are the factors that either encouraged or discouraged parents and adolescents in communicating SRH information. A person can be encouraged to seek for information to communicate, and a person with information can be encouraged by it to communicate. Three subthemes emerged here: parent factors, adolescent factors and the parent-adolescent relationship.

#### Parent factors

This refers to the parents’ favourable or unfavourable evaluation of their SRH information communication with their adolescents. In this study, the parental factors were the parent’s attitudes towards SRH information communication, discerned threat, the educational background of the parent, their occupation and the cues for communication.

Parents believed that communicating SRH information is important because adolescents can get misinformed from the wrong sources, which may be devastating. This parent therefore considered himself as the right source of information:

‘It is very important because if you don’t share the right information they may be misinformed. They may get it from the wrong source and in the end, it will not help them, so they need to get it from somebody who has knowledge and can guide them.’ (P9, M, 63 years)

Parents also felt it to be their duty and a God-given mandate to raise their adolescents to have a better future and therefore it was expedient for them to communicate on SRH matters. They use the Bible as their guide to train the adolescent. They also did not want the fees they pay to educate their adolescents in school to go to waste, knowing that teenage pregnancy and its consequences can hold the adolescent back. A participant shared that:

‘Personally, I know God has given me this girl and it is my duty to train her to be a good adult. So, I make sure that all that the Bible has said concerning that we train the child the way he should be, so that when he grows, he will not depart. I make sure I instill discipline in her. The fees … I don’t want it to go waste, when you get pregnant, things are going to go bad.’ (P6, F, 36 years)

When parents were not willing to share information, it was not because they believed that communicating SRH information to adolescents was bad in general, but because of their perception about some of the topics. Once they had discussed abstinence, they felt there was no need to talk about topics such as contraceptives and abortion, because once an adolescent abstains from sex, there will be no need for contraceptive usage. A participant shared that:

‘Contraceptives …, I didn’t … like I said, if you’re not ready for marriage, don’t indulge in sex. I try not to let any of them know that there is something to prevent pregnancy, so all I talk about is abstinence. Abortion, I don’t remember talking to them, in fact abortion actually didn’t even cross my mind … my interest was abstaining from sex.’ (P9, M, 63 years)

Some parents felt that discussing certain topics would expose the adolescent to practicing risky sexual behaviours, so they were afraid to share these topics with the adolescents. For instance, a parent shared that:

‘I felt like it is too much for her to get that lot of information, because sometimes if you have such information, you intend to practice it; so, I was afraid to go there.’ (P7, M, 40 years)

Other parents believed that information would rather empower the adolescents to take decisions. They felt it is the older generation who hold the idea that exposure to information leads to practice. A parent argued that:

‘I think it’s okay, but our older generation … they feel the more you expose, we’re endangering the young, the adolescent, instead of making them better … but for me it is the reverse: the more you know, the more power you have to be able to help yourself.’ (P8, M, 48 years)

Parents had fears that, without communication, adolescents might indulge in risky sexual behaviours and experience the consequences, such as teenage pregnancy. Such fears encouraged them to search for information and communicate it with their adolescents:

‘You are always afraid, because in the community, you look around and you see teenage pregnant girls and the way they are suffering. So, I don’t want my daughter to be part. That is why I took a lot of effort to do research, do a lot of reading to impact my child.’ (P7, M, 40 years)

It was also found that the educational level of a parent could influence SRH information communication. Parents stated that those who are educated are exposed to information on SRH and tend to share their knowledge of it with their adolescents. A participant mentioned that uneducated parents do not care about their adolescents:

‘I think the educated ones are rather getting information or helping to build up their children, but the uneducated ones don’t care about their kids.’ (P5, F, 39 years)

The occupation of the parent was also cited as a possible facilitator for SRH information communication. For instance, a teacher mentioned that the subject she teaches has equipped her to communicate with her adolescents on SRH issues:

‘I teach Religious and Moral Education, and I teach all those things in the school. When I reach that place, I don’t hide anything from them, so I’m not afraid or shy to talk to my children about it.’ (P5, F, 39 years)

Parents also wanted to look for some signs of the adolescent’s interest in the opposite sex, or tendencies to engage or learn about sexuality before they initiate communication. These were cues that led to communication. A parent shared that:

‘I wanted to see … the tell-tale signs. I started looking for evidence to see whether they are showing signs of being interested in the opposite sex, and if I didn’t find one, I leave them until when I saw that surfacing … I started talking to them on sexuality.’ (P9, M, 63 years)

#### Adolescent factors

These were the characteristics of adolescents that either encouraged or discouraged parents from communicating SRH information with them. The adolescent’s willingness to communicate with the parent and the stage of adolescence were factors that were identified in the study which either encouraged or discouraged the parents from communicating.

A parent noted that, at a point, adolescents were not comfortable with communication about sex, so she had to pause until a later date. However, adolescents were comfortable with topics such as menstruation. Parents said this is because, in the Ghanaian context, it is difficult talking about sexuality:

‘In our part of the world, it is difficult to talk about sexuality, so when I started, she was comfortable with the menstrual cycle activities, but when the sexual activities came in she was very uncomfortable, so I had to break, and continued later.’ (P7, M, 40 years)

Another parent shared that her adolescent was not willing to participate in the communication because she was not comfortable with sex-related topics. She felt she did not involve herself in sex, and that listening to such information could cause them to practice:

‘It is the eldest one that I used to talk to about sex, but she won’t even allow you. She will say since she is not doing it [*having sex*], I should not let it come in her mind, for her to do it.’ (P1, F, 38 years)

Parents also realised that adolescents may not be in a good mood when they may be ready to speak to them:

‘But we only talk about them when she is in a good mood.’ (P10, F, 50 years)

The stage of the adolescence was another factor that was mentioned by parents which influenced the communication. Parents stated that the content of the communication depends on the stage of the adolescent. A parent mentioned that in early adolescence, certain topics such as sex, abortion and contraceptives are not to be discussed. He would rather wait for when the adolescent is at the secondary school, at which point he feels the adolescent will be matured and exposed and have more desire to have sex, before he will focus on discussing such topics:

‘As I said she’s still in her early adolescence … so sex, abortion, contraceptives are out of our discussion. Possibly at secondary school where she will be more matured, I can upgrade the conversation better as she prepares for tertiary education, where the trends and other things will be more, the threats, the temptations and the desire will be more.’ (P8, M, 13 years)

The above information highlights that parents expect SRH information to be categorised according to the stage of the adolescent. This can be taken into consideration in developing an intervention or guidelines to facilitate SRH information communication.

#### Parent–adolescent relationship

The findings revealed that when parents have a good relationship with their adolescents, it helps them to communicate SRH information. A parent mentioned that she can talk to adolescents without restrictions when she plays with them and creates an atmosphere where the adolescent will be bold to ask questions. She mentioned that strictness does not permit the adolescents to flow when communicating with their parents:

‘It is because of the relationship I have with her. If you are free with your kids, you can talk to them without any restrictions. But if you are strict, it will be difficult talking to them. If you even talk to them, they will find it difficult to ask questions. But because of how I joke and play with them we are able to talk freely.’ (P5, F, 39 years)

### Contextual factors influencing sexual and reproductive health information communication

These are the parent’s perceptions of social support from significant others and the behaviour of people in the community that influences SRH information communication between parents and their adolescents. Two subthemes emerged, which are perceived social support and cultural norms.

#### Perceived social support

This refers to the parents’ perceptions of the support available from their significant others for communicating SRH information with adolescents. The opinions of significant others contributed either positively or negatively to SRH information communication. In the study, a level of support from significant others was identified. Participants believed that social support has improved and that this emanates from the fact that SRH has gained attention currently and there are events that are organised in the communities and on the radio stations to talk about SRH issues:

‘I think up to a level, not to the extreme but up to a level, at least the number of events and activities in our societies, in our community touching on them [*SRH information*] and also radio.’ (P8, M, 48 years)

A participant shared that their significant others are divided in terms of support for SRH information communication with adolescents. Some family members positively evaluate it while others are negative about it.

‘The family is divided. Some think it is important, others think it is not good to talk about sex to your child. So, the family is divided.’ (P4, M, 42 years)

#### Cultural norms

Culture is the social behaviour of a particular group of people in a society. These norms were identified as influencing the way parents communicate to their adolescents regarding SRH.

Mentioning the sexual organs in the local language has been described by parents as a hard thing to do. Parents mentioned that their parents did not communicate much about sexuality and that has made it difficult for them to also communicate. This identifies the cultural influence on the communication process. It was also noted that some parents see it as a taboo to talk about sexuality. A parent mentioned that:

‘Using the local language for our private parts is like a heavy thing. Our forefathers and our parents didn’t want to talk about it. That’s why some parents think it is a taboo to talk about it, especially using the local language to call some sexual organs.’ (P4, M, 42 years)

Parents who mention sexual organs in communication with adolescents are considered bad, so the communication has to be proverbial. A parent shared that:

‘In our culture, there are some parts of the human body, sexual parts, you can’t mention them. You have to speak about it proverbially. If you speak too straight about it, you are seen to be a bad person.’ (P8, M, 48 years)

Another parent shared that he has been raised to believe that talking about sexuality is inappropriate. He mentioned that cultural background can influence one’s view of things. He shared his experiences when growing up and how difficult it was to talk about sex. He mentioned that one could be punished for talking about sex.

‘We have been raised to believe that talking about sexuality is inappropriate, and how our culture has been structured … I remember when we were young, you can’t talk about sex issues with your parents. You’ll be shut by your parents. Some people even go as far as receiving punishments. But I want to protect my children.’ (P7, M, 40 years)

The parent shared that he wants to discuss sex with his adolescents because he believes that the knowledge will rather protect the adolescent. This is suggestive that, although culture has a role, a person’s motivation to comply with communication is important.

### Sexual and reproductive health communication skill needs of parents

This is the way in which parents share SRH information and the skills required for that communication with adolescents. Two subthemes emerged: actual ability to communicate and nature of communication.

#### Actual ability to communicate

This is about a parent’s ease and comfort in communicating SRH information, its frequency, and the belief that he or she will be able to sustain communication on SRH with the adolescent.

Parents shared how comfortable they feel when communicating SRH information with their adolescents. A parent shared that she has the skill to talk with the adolescent freely. She considered the communication as a normal one which is not strict and comes freely. A parent shared that:

‘We are free, we talk, we laugh, so it is normal conversation. It is not something strict. I don’t sit her down strictly, no no no; we just communicate as we have been doing always.’ (P5, F, 39 years)

Parents used various strategies to communicate with their adolescents. They intentionally created an atmosphere to facilitate the communication:

‘But when we are all happy, chatting, laughing, then I bring those things [*sexuality*].’ (P9, M, 63 years)

Some parents were able to sit or lie down comfortably with their adolescents to talk about SRH matters.

‘We were lying down on the bed, comfortably lying down on the bed. It was like a conversation.’ (P10, F, 50 years)

Another participant had built the skill of talking to the adolescent in an atmosphere of calm. Being a guardian, staying with the adolescent for years, had led to the development of a bond that fostered communication:

‘Nothing makes it difficult for me to talk with her. I talk nicely with her, I don’t shout at her.’ (P3, F, 42 years)

Another participant shared how she uses scenarios and experiences to communicate SRH information with her adolescent:

‘I use scenarios and things that I come across … experiences to explain things to her.’ (P6, F, 36 years)

A parent also shared that persistent communication has made it easier for him to communicate SRH information, and therefore, going forward, he believes in his ability to communicate. The perceived self-efficacy was noted in this parent’s view:

‘Now it is easier, because I’ve been able to jump the first part, so I will be able to open up to her and she too will open up to me in our conversations. Moving forward, I don’t think I will have that feeling when I was talking to her in terms of sexuality at first.’ (P7, M, 40 years)

This indicates that a skill can be built from experience, although it can also be facilitated by training and empowerment.

#### Nature of sexual and reproductive health information communication

This concerns how SRH information was communicated with the adolescents. The difficulties faced by parents in communicating with their adolescents highlighted the skill needs of parents. It was noticed that initiating SRH information communication was difficult for parents and was coupled with thoughts of how the adolescents would receive the information. They were, however, motivated to communicate because they felt it is a responsibility. A parent stated that:

‘Sometimes to start with it is very difficult. When I want to talk about sexual matters, it’s difficult, but I try because I see it as my duty … so starting it is difficult, but I try. Sometimes, I think about how she is going to take it. But when I start, she responds to the question, and we discuss.’ (P4, M, 42 years)

This implies that beginners strongly need assistance in building SRH communication skills. Parents and adolescents may lose interest in further communication if the initial one does not go well.

The nature of SRH information communication also highlights the communication skill needs of parents. Some parents felt that they had to find a way of convincing adolescents not to involve themselves in sexual activities. One participant, for instance, recounted how she had to make it look horrible to deter her adolescent from indulging in sexual behaviours:

‘So, I made it look horrible so that she will not go near it.’ (P10, F, 50 years)

Parents resorted to any means available, such as begging or warning, to get SRH information across to the adolescents. A participant shared that:

‘We were talking about pregnancy, and I even begged her not to be involved in sex, because if she does, it is going to affect her education.’ (P4, M, 42 years)

‘I have warned you, don’t be walking with women. I’ve told you that this boy, I don’t like him coming to my house with you, or some kind of things.’ (P9, M, 63 years)

A parent may be willing to communicate but may not have the skill to do it well. In general, the communication between parents and adolescents came with challenges, which define the communication skill needs of parents. This means that parents need to be taught the approaches they can take to effectively communicate with their adolescents on SRH matters.

It was found that SRH information communication was not an activity which was usually practiced among parents. A parent mentioned that she had not been communicating SRH information frequently:

‘To be honest it isn’t frequent, I have done it twice.’ (P7, M, 40 years)

‘The last time we talked about sexuality was about a year ago, if not more than a year.’ (P10, F, 50 years)

This highlights the need for a culturally appropriate SRH information communication intervention to train parents and adolescents on how to communicate SRH information.

## Discussion

**FIGURE 2:**
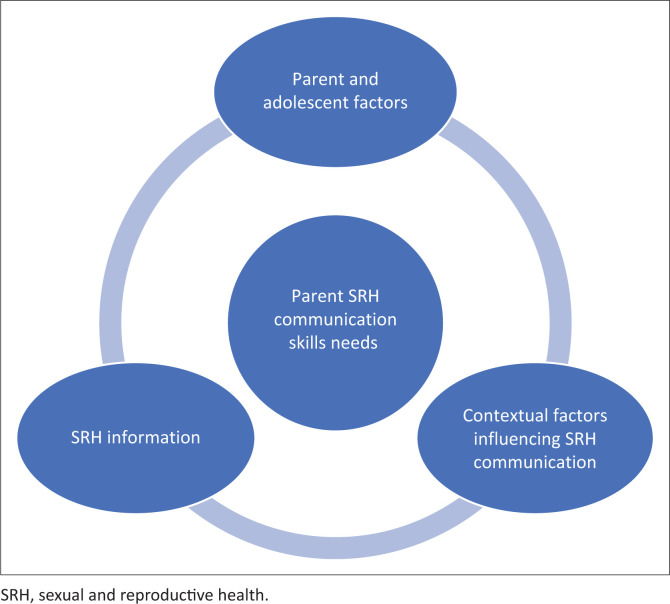
Sexual and reproductive health communication skill needs of parents as they emerged from the study.

This study has highlighted that SRH information of parents, individual parent and adolescent factors and contextual factors influence the communication skill needs of parents. The three factors that influence the SRH communication skill needs are related to each other; the parent and adolescent factors influence the kind of SRH information to seek and the kind of information determines how well one can share it as parents were more comfortable sharing information on some SRH topics than others. This has been summarised in Figure 2.

### Sexual and reproductive health information communicated

Parents reported discussing changes in adolescence, menstruation, personal hygiene, abstinence, teenage pregnancy and its consequences, sex, sexual relationships, STIs and virginity with their adolescents. Some other studies in Ghana have reported similar findings regarding the range of topics discussed.^27,28,29^ This is also consistent with other studies in Africa, specifically in Uganda^30^ and Nigeria.^4^ The similar findings in these studies could be attributed to the strong sociocultural and religious norms across Africa. This understanding is supported by a study in the United States which revealed how parents support the extensive discussion of other topics such as sex and contraceptives, while these were among the topics least discussed in the current study.^31^

The topics most discussed in this study were menstruation and abstinence. This finding corroborates findings of other studies.^29,32^ This is typical of African culture, in which sex is not usually discussed because of its perceived tendency to introduce adolescents to practice and because of how sacred it is.^33^ Parents desire their adolescents to live a chaste life and avoid the negative consequences of adolescent involvement in sexual activities.^34^

This study also gives insight into the various sources of SRH information. The sources identified included books, workshop materials, radio, television, internet, phones, social media, church, mosque, school, workplace and personal experiences. A similar range of information sources has been mentioned in previous studies.^35^ These sources were mentioned because they are probably the most available sources. Those who were not educated could not use the social media for information but mostly relied on their personal experiences and what friends have shared. This was also reported in other studies.^29,36^ While experiences and learning from others was a source cited, it is worth noting that, if a person with inaccurate knowledge is consulted, inaccurate knowledge will be transferred. It should be noted that accurate information will depend on the source. However, evidence suggests that parents are aware of this gap and are willing to undergo training to improve their knowledge of SRH and communication of the information.^9^

### Parent and adolescent factors

It was found that parents have positive attitudes towards SRH information communication. This builds on earlier studies that have reported that parents have positive attitudes towards discussing SRH topics with their adolescents.^10,37,38^ Their positive attitude towards SRH information communication cannot be generalised to include all topics under SRH, as parents did not discuss certain topics. However, it is worth noting that parents with positive attitudes towards SRH information communication are more likely to communicate than those with negative attitudes.^39^

Parents also had fears that lack of communication of SRH information with their adolescents may lead to adolescents indulging in risky sexual behaviours and experiencing the consequences. This is in line with other study findings that highlighted the influence of perceived threat in increasing SRH information communication between parents and their adolescents in Nigeria^4^ and Kenya.^40^

The corroboration of the findings in all these studies highlights the fact that parents have expectations of their adolescents and would like to communicate to avoid the unexpected.

The identified demographic factors–influenced communication in the study included the age of the adolescent, the educational background of the parents and their occupation. Similar results have been observed in Ethiopia, where the age of the adolescent and the education level of the parents were cited as factors influencing SRH information communication between parents and adolescents.^41^ This also affirms what has been identified earlier in Nigeria^42^ and in a review of studies about parent–child communication about sexuality in sub-Saharan Africa.^43^ This suggests that parents without a formal education may find it difficult to discuss SRH information. This being the case, training in making use of an appropriate intervention will empower such parents to meet the SRH needs of their adolescents. It was also found that parents wanted to wait until adolescents mature cognitively or have reached puberty before initiating communication. This was also in line with other study findings that noted that parents waited till puberty to initiate communication on SRH issues with adolescents, because of the belief that adolescents were innocent before puberty.^44,45^

Parents waited to see certain signs before they could initiate communication on SRH issues. This is congruent with the findings of a study on home-based sexuality education in Ghana.^28^

It was also found that the relationship between parents and their adolescents plays a critical role in SRH information communication. This was in line with a study in Tanzania^15^ which identified the parent–adolescent relationship as a facilitator of parent-adolescent SRH information communication.

### Contextual factors

It was found that subjective norms influence SRH information communication by parents. This result supported that of other research which found that significant others have influence on parent-adolescent communication on sexuality.^28,38^ A level of social support was identified in the study, but according to participants it was limited with regards to the topics to be discussed, taking into consideration also the age of the adolescent. This confirms the earlier submission that parents considered the age of adolescents before accepting to discuss SRH issues.^46,47^ In all, when the topics are menstruation and abstinence from sex, significant others are more likely to support the communication. This could also be due to the strong cultural backgrounds of these significant others. That means that any intervention that does not consider these norms may be rejected and not be of use to parents and adolescents.

Participants in this study mentioned how their cultural norms do not support the open discussion of SRH issues. It was not common to mention the names of the sex organs in the local dialect and parents had to use euphemisms. This was also indicated in other studies in Ghana and Ethiopia.^5,9^ Therefore, it could be said that an intervention that is culturally appropriate is what is likely to be accepted by parents and adolescents to guide them in communication.

### Sexual and reproductive health communication skill needs

Although some parents in the study indicated that they can communicate with their adolescents on SRH, it came with challenges. Parents who mentioned that they could easily communicate had confidence in some limited topics but had skill needs. This affirms an earlier study in Ghana^29^ which noted that parents had some difficulties communicating SRH information with their adolescents. This highlights the fact that the communication skills in general need to be sharpened through training. Studies have identified lack of communication skills to be a major contributor to parents not discussing SRH issues with adolescents.^28^ These challenges could be because of the technical nature of SRH terminology, as well as social and cultural norms. It was also found in the current study that some of the strategies used by parents were to warn adolescents about sex and its consequences. These highlight the need for parents and adolescents to be trained using a culturally appropriate intervention. Parents put fear in the adolescents as a strategy to have them stick to the advice they gave, to prevent pregnancies and STIs. A study reported similar findings in Nairobi, Kenya.^40^ However, this approach has been perceived to be positive in influencing behaviour^48^ and in preventing pregnancies in some cases.^16^

### Strengths and limitations

The diversity of ethnic groups in the study is a strength in understanding how culture influences the communication process. The setting contains an influx of migrants from both urban and rural communities, which makes the findings not particularly skewed to either urban or rural settings.

The researcher ensured that preconceptions of cultural and professional affiliations did not influence the data interpretation. Reflections and supervisory guidance were maximised in the data interpretation.

Although the findings have important theoretical and practical implications for the adaptation of a culturally appropriate SRH information communication intervention, there are, however, some limitations that are worth mentioning in interpreting the findings. The study was guided by the IMB skills model and so the limitations of the model, such as environmental and cultural factors which are not part of the constructs, could impact the study findings. However, the inductive approach which was employed in the analysis was able to control this to some extent. Most of the participants were educated to the tertiary level, and that might have impacted on the findings, which must therefore be interpreted with caution. The parents selected had adolescents between 13 and 16 years of age, and so parents’ communication experiences may not be generalised to parents of adolescents above this age, although similar experiences might have been observed when those adolescents were within the age range of adolescents for this study.

## Conclusions

The research highlights the SRH information communication skill needs of parents. Sexual and reproductive health communication skills depended on the information parents had of SRH, the individual parent and adolescent factors and contextual factors. Parents experienced a tension between their culture and their need to communicate SRH information to their adolescents. There is a need for guidance in initiating communication and there is also a need for parents to know the approaches which can be used to communicate SRH information effectively. It can be concluded that an adoption of a parent-adolescent SRH information communication intervention, which is culturally appropriate, to train parents and adolescents may help meet these communication skill needs of parents to empower them to communicate SRH information with their adolescents.

### Recommendations

It was noted that an adolescent’s readiness and willingness, as well as skills for communication, are important to assist parents to effectively communicate SRH information with them. Therefore, it is recommended that adolescents’ SRH information communication skill needs in Ghana are explored qualitatively by SRH researchers to understand how adolescents’ experiences influence SRH communication with their parents. There is also the need for further studies to focus on the adaptation of a culturally appropriate parent-adolescent SRH information communication intervention, to train parents and their adolescents on communicating SRH information.
